# BA-CD Composite Polymers for Efficient Adsorption of Diverse Dyes and Its Mechanism: A Discussion-Based Thermal Dynamic and Kinetic Study

**DOI:** 10.3390/polym17172357

**Published:** 2025-08-29

**Authors:** Zhaona Liu, Make Li, Yangyang Zheng, Huacheng Zhang

**Affiliations:** 1 Department of Pharmacy, Medical School, Xi’an Peihua University, Xi’an 710125, China; zhaonaliu@peihua.edu.cn; 2 School of Chemical Engineering and Technology, Xi’an Jiaotong University, Xi’an 710049, China

**Keywords:** BA-CD, macrocyclic polymers, cationic and anionic organic dyes, absorbent, adsorption

## Abstract

Boric acid/β-CD-based polymers (BA-CD) possess hierarchical porous structures and efficient functional groups for further molecular recognition, which are used for the adsorption of a series of cationic and anionic organic dyes. The effects of pH, contact time, initial concentration of solution, and temperature on the adsorption performance were experimentally investigated in detail. Surprisingly, the adsorption capacities of BA-CD towards RB exhibited a higher value of 733.2 mg g^−1^ among a series of cationic and anionic dyes. The adsorption kinetics further indicated that the adsorption of dyes by BA-CD belonged to a quasi-second-order kinetic model, while the adsorption isotherms demonstrated the adsorption process as the Langmuir isotherm model. The characterization of the adsorption process was performed in the presence of monomolecular layer chemisorption. In addition, the reusability test showed that BA-CD had a high reusability rate of 90% in MG after five cycles, indicating its future potential for the treatment of dye wastewater.

## 1. Introduction

With the development of industry, pollution-related problems such as the treatment of polluted water have dramatically arisen in the natural environment and become an important focus from both public and scientific perspectives [1]. In particular, dye wastewater accounts for a relatively high proportion of polluted water types [2,3,4,5] as industrial applications are progressively discharging huge amounts of it [6,7,8,9,10]. It is greatly affecting both the health of humans and the survival of human communities on our mother planet—the Earth. Adsorption is a general, convenient, and green strategy for treating wastewater among all physical, chemical, and biological dye wastewater treatments in the past decades [4,11,12,13,14], and the success of adsorption heavily depends on the specialized materials employed. Thus, the development of new cheap, effective, and sustainable functional adsorbents has become a key issue in both academic research and practical engineering studies in carrying out wastewater treatment to remove targeted pollutants [12,13,14,15,16,17].

Organic and polymeric materials, matrix, and frameworks are usually synthesized efficiently by the direct one-pot method, thoroughly investigated as potential candidates for adsorbents [18,19,20,21,22,23], and continuously developed for a wide range of applications to adsorb diverse “targets” [24,25,26]. Various functional molecules and monomers are involved during the synthesis of these kinds of materials, for example, boric acid (BA) [27,28,29] is a significant building block as a crosslinking agent in preparing a covalent organic framework (COF), and a basic ingredient to prepare the spatially limited porous matrix for accommodating specialized fluorescent dyes to accommodate room temperature phosphorescent materials with long afterglow phenomena [30]. But how to lower the cost in large-scale preparation [31,32], how to efficiently differentiate and confirm boric acid’s complicated structures at the molecular level, and how to generate hierarchical diverse porous structures [33,34] to enable the adsorption of a wide range of targeted pollutants are still debatable in extending the practical applications of these kinds of organic and polymeric materials in industrial areas.

Consequently, researchers from both organic synthesis and materials science have attempted to address these problems by employing multiple solutions, for example, introducing macrocyclic molecules [35,36] in these organic and polymeric materials to promote the formation of hierarchical pores. It is well known that the commercially available and environmentally friendly macrocycle β-cyclodextrin (β-CD) can form specialized host/guest [37,38,39] inclusions by capturing its favorite guests/pollutants due to its unique truncated cone-shaped structure and hydrophobic cavity [40,41,42].

To develop low-cost, effective, and sustainable functional adsorbents, we previously combined the convenient one-pot synthetic strategy [30], the valuable crosslinking building block of BA, and the attractive macrocyclic architecture of β-CD, producing a new macrocyclic polymeric material, BA/β-CD-based polymer (BA-CD) [43,44,45,46]. We have comprehensively proved that such polymeric composites have specialized monomeric structures at the molecular level, with BA as the bridge connecting CD moieties together, and the synergistic effect of neighboring macrocyclic cavities in this kind of polymeric material enhanced molecular recognition of different-sized guests with various ionized properties [46].

With the study of synergistic effect in molecular recognition by BA-CD having been established, here, we would like to further employ this amorphous BA-CD [30,43,44,45,46] in the adsorption of different cationic and anionic organic dyes, focus on detailed parameters in the process of adsorption, and expand its application to model the situation in the treatment of industrial water with dyes. Thus, the effects of single-factor conditions on the adsorption performance were systematically analyzed in detail. And the performance of BA-CD was fully experimentally confirmed for the efficient capture of a series of dyes such as tartrazine (TZ), direct red 28 (DR), crystal violet (CV), rhodamine B (RB), malachite green (MG), and methylene blue (MB).

Additionally, the molecular-level mechanism behind the good performance in adsorption should not only be attributed to the cooperation between neighboring β-CD moieties, as mentioned in our previous studies [46]. If only the β-CD moiety can be considered during the adsorption process, why should we build the aforementioned hierarchical structures? In other words, will those in situ generated pores bridged by BA also participate in the adsorption? If so, how much do they contribute during the adsorption? And is there any additional evidence to support the synergistic effect between CD cavities and in situ generated pores? Thus, thermal dynamic studies were further carried out here, revealing the driving force and possible cooperation among these hierarchical pores in accordance with the analysis of size and structural issues in the pores. And studies of adsorption kinetics and adsorption isotherms were then carried out to prove that both physical and chemical adsorption models performed well in the adsorption of dyes by BA-CD.

## 2. Materials and Methods

### 2.1. Materials and Instruments

The MB (>98%), MG, CV, DR, and TZ were all purchased from Macklin (Shanghai, China) without further purification. And the RB was purchased from Tianjin Heowns Biochemical Technology Company. Ultrapure water was directly made in the lab by an Ultra-pure water system. The powder polymeric BA-CD was prepared by using the reported method, and was fully characterized and confirmed according to the same physiochemical properties as mentioned in the literature [30,45,46]. A UV-vis absorption spectrometer (TU-1901 Beijing Pukinje GENERAL Instrument Co., Ltd., Beijing, China) was used to determine the concentration of residual dyes in the supernatant by measuring the standard curves at the maximum wavelengths of each type of dye.

### 2.2. Determination of Dye Solutions’ Standard Curve

Standard solutions (2.0, 4.0, 6.0, 8.0, 10.0, 15.0, 20.0, and 25.0 mg L^−1^) of each dye in a concentration range of 2.0–25.0 mg L^−1^ were prepared. The absorbance of the solutions was measured at the maximum wavelength of the dyes. The concentration of each solution (C, mg L^−1^) was taken as the horizontal coordinate, and the absorbance (Abs) was taken as the vertical coordinate to fit the standard curves.

### 2.3. Single Adsorption Performance Studies

In a 100 mL conical flask, 30 mL of dye solution was added, and then 10 mg of adsorbent was added. The top of the flask was sealed with cling film, and further placed in a constant temperature oscillator. It was oscillated at a certain temperature at an oscillation rate of 130 r min^−1^ until the adsorption reached equilibrium. The subsequent experiments were carried out at pH = 7. The concentration of the adsorbed dye was determined using UV-vis and the remaining concentration of the solution was determined from the standard curve. The adsorption capacity of the adsorbent for the dye, q_e_ (mg g^−1^), and the removal rate, R%, were calculated by Equations (1) and (2) [43,44].

(1) $$q_{e}=\frac{(C_{0}-C_{e})V}{m}$$

(2) $$R\%=\frac{C_{0}-C_{e}}{C_{0}}\times 100\%$$

where C_0_ (mg L^−1^) and C_e_ (mg L^−1^) are the initial dye concentration and the dye concentration at equilibrium time, respectively, and V (L) and m (g) are the volume of the dyes and the weight of the adsorbent, respectively.

## 3. Results and Discussion

### 3.1. Effect of Absorbance on Adsorption Performance

**Figure 1:**
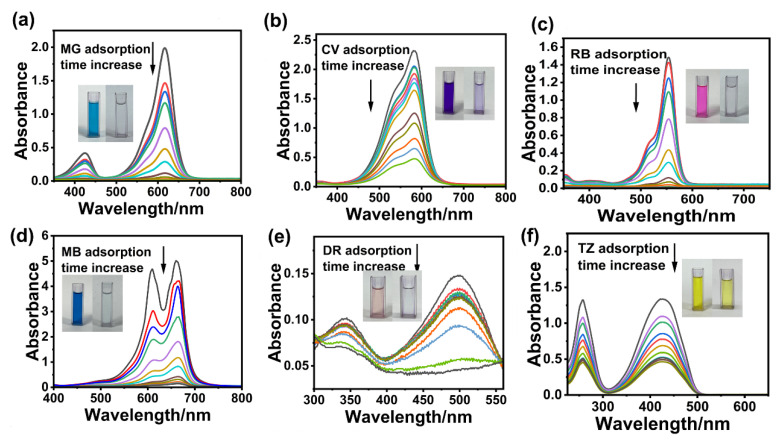
(**a**–**f**) Effects of absorbance on the adsorption properties of MG, CV, RB, MB, DR, and TZ. The absorbance deceases in according to the increase of adsorption time.

**Figure 2:**
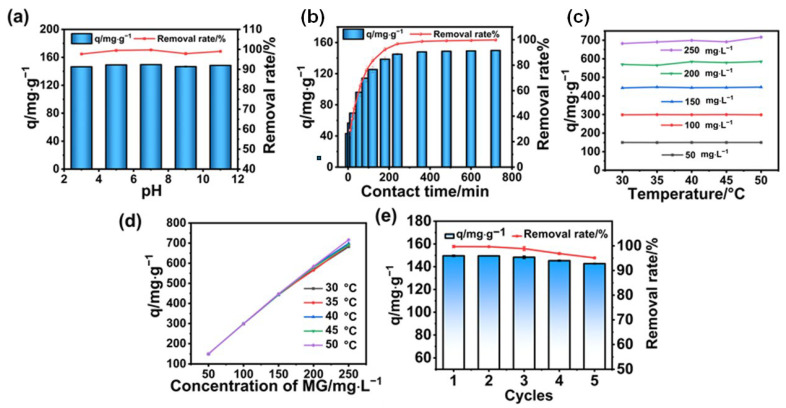
Adsorption performance of MG, revealed by (**a**) pH effect, (**b**) contact time effect, (**c**) temperature effect, (**d**) concentration effect, and (**e**) cycle effect.

To precisely investigate the adsorption performances of the individual dyes, the effect of absorbance on adsorption performance was initially studied by determining the change in concentration of each dye. When the adsorption time increases, the remaining concentration of the MG dye decreases gradually, as revealed in Figure 1a. When reaching adsorption equilibrium, the dye concentration approaches zero. The inset image of Figure 1a is a photograph of the solution before and after the adsorption of MG by the BA-CD adsorbent. The obvious color changes from blue to almost transparent, indicating the high adsorption effect of the targeted adsorbent. The absorbance changes of the other five dyes tend to decrease in dye concentration in accordance with time (Figure 1). In the photographs of the dyes before and after adsorption as shown in the inset image of Figure 1, the dye color is obviously lighter. Thus, the adsorption of dyes by BA-CD was successfully evaluated by the change in absorbance. Based on this detection of adsorption performance, further investigations including pH effect, contact time effect, temperature, concentration effects, and regeneration cycles were carried out. Figure 2 presents a typical example of each of these experiments, comparing aspects of different adsorption performances of BA-CD in relation to MG.

### 3.2. Effect of pH on Adsorption Performance

The magnitude of solution pH also had an important effect on the adsorption of each dye. First, the maximum adsorption of MG by BA-CD reached 149.6 mg g^−1^ at pH 7 (Figure 2a). In fact, the adsorption capacity of the adsorbent for MG did not change much when the pH increased from 3 to 11, which might be caused by the unique property of this adsorbent. We suppose that because negatively charged groups are embedded into the surface of the adsorbent, the adsorption performance is consequently not much affected by pH after the cationic dyes have been combined with the negatively charged groups.

**Figure 3:**
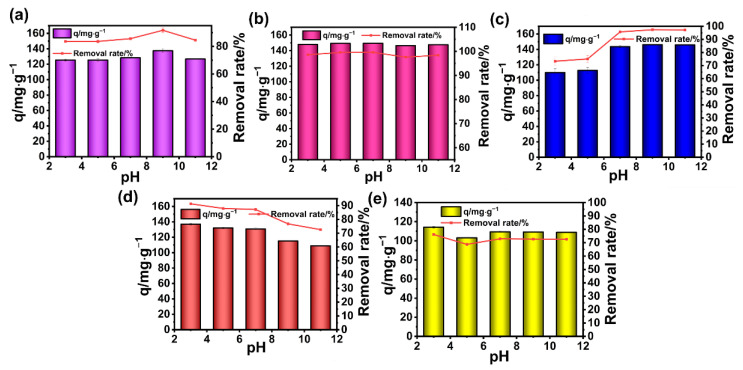
Effects of pH on the adsorption properties of CV (**a**), RB (**b**), MB (**c**), DR (**d**), and TZ (**e**).

The effect of pH on RB followed a similar trend as shown in the case of MG, and did not change much. In particular, the maximum adsorption capacity of 149.4 mg g^−1^ towards RB was achieved at pH 7. In other cases of cationic dyes such as MB and CV, the adsorption capacity of BA-CD increased in accordance with the increase in pH (Figure 3), finally reaching 146.1 mg g^−1^ and 137.5 mg g^−1^, respectively. This might be caused by the presence of more protons in the water solution at lower pH values, which significantly competes with the cationic dyes for recognition by adsorption sites on the adsorbent, leading to a clear decrease in the efficiency of the adsorption. By contrast, for two additional anionic dyes such as DR and TZ (Figure 3), the maximum adsorption capacity of 137 mg g^−1^ and 114.2 mg g^−1^ by BA-CD was achieved at low pH values. Thus, dye solutions with different pH values did influence the performance of BA-CD in adsorption (Figure 3).

### 3.3. Effect of Contact Time on Adsorption Performance

To determine the effect of contact time on the adsorption performance of BA-CD, the investigation was carried out at an initial dye concentration of 50 mg L^−1^. The adsorption capacity for MG increased rapidly within 240 min, and the adsorption efficiency reached 96.73%, owing to the abundance of unoccupied active sites on BA-CD during the pre-adsorption period (Figure 2b). Then, more MG molecules gradually occupied the adsorption sites. When the adsorption gradually tended to the equilibrium, the maximum adsorption capacity of 149.8 mg g^−1^ was reached at 720 min and the removal rate was 99.87%.

**Figure 4:**
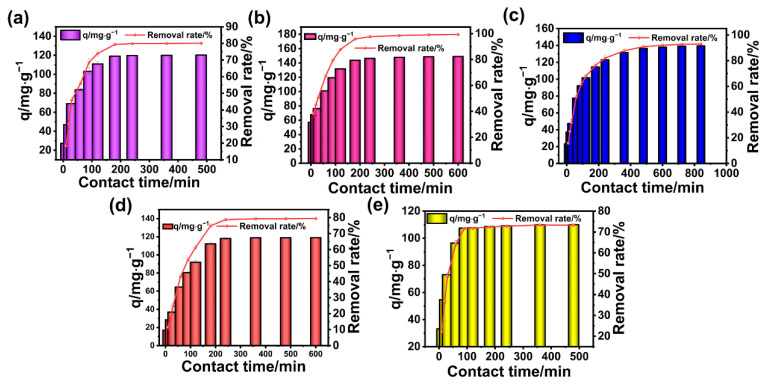
Effects of time on the adsorption properties of CV (**a**), RB (**b**), MB (**c**), DR (**d**), and TZ (**e**).

This is the same as the results shown in Figure 4, except that the time point of reaching adsorption equilibrium and the adsorption capacity of each dye were different. In particular, for DR and TZ, the time to reach the adsorption equilibrium was faster. But it takes more time for MG, CV, RB, and MB to reach the adsorption equilibrium. Additionally, they had a higher adsorption capacity than DR and TZ. Thus, BA-CD adsorbs cationic dyes better, due to the large number of negatively charged groups on its surface for molecular recognition of cationic dyes (Figure 4).

### 3.4. Effect of Concentration of Dyes on Adsorption Performance

To determine the different adsorption performances of BA-CD at diverse concentrations, the investigation of its adsorption of MG with different concentrations was initially carried out. As expected, the adsorption capacity of BA-CD increased in accordance with the increase in concentration of MG. For example, the adsorption capacities of BA-CD at 30 °C for MG at 50 mg L^−1^ and 250 mg L ^−1^ were 149.6 mg g^−1^ and 682.2 mg g^−1^, respectively (Figure 2c).

**Figure 5:**
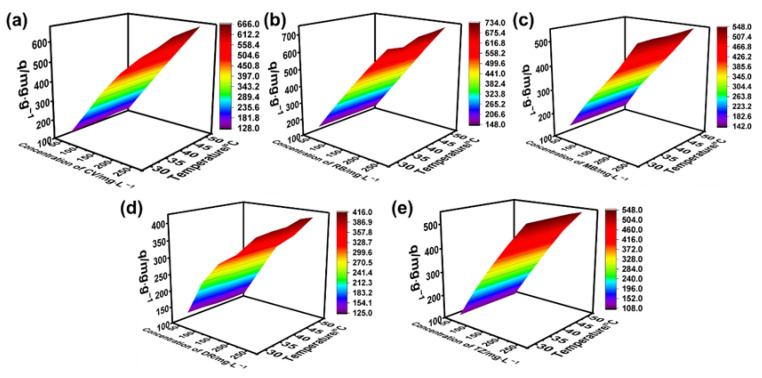
Effects of concentration and temperature on the adsorption properties of CV (**a**), RB (**b**), MB (**c**), DR (**d**), and TZ (**e**).

**Figure 6:**
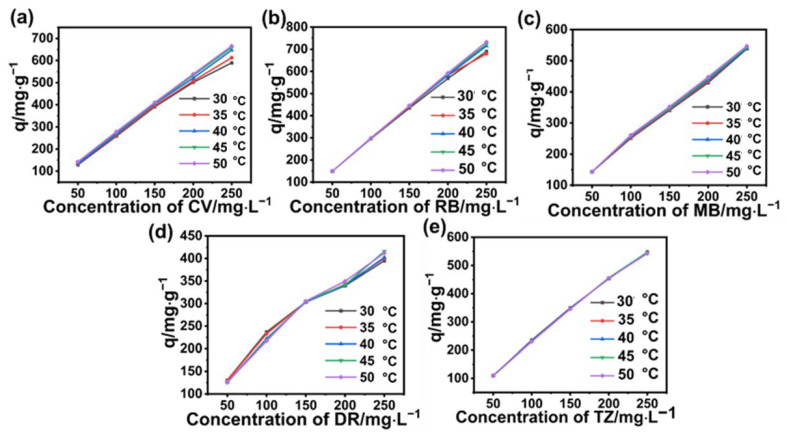
Effects of concentration on the adsorption properties of CV (**a**), RB (**b**), MB (**c**), DR (**d**), and TZ (**e**).

The significant enhancement in adsorption capacity is attributed to the high concentration of MG in solutions. As known, when the solution was driven by the large concentration gradient, mass transfer between solid and liquid could be enhanced. Thus, solutions containing a high concentration of dyes are ideal for adsorbents. Furthermore, it was found that increasing the temperature of the solution also affects the adsorption capacity, i.e., the higher the temperature, the higher the adsorption capacity, indicating that the adsorption process favors high temperature. The situations of the other five dyes also revealed the same result, i.e., the adsorption capacity of BA-CD increased almost linearly in accordance with increasing the concentration of dyes (Figure 5 and Figure 6).

### 3.5. Effect of Temperature on Adsorption Performance

Temperature always has different effects on the diffusion rate of diverse molecules, which can further affect the adsorption performance of the adsorbent [47,48]. To figure out the temperature issue, MG was then chosen as the first target dye for adsorption tests by BA-CD. In accordance with the temperature rising, the adsorption capacity of BA-CD on MG in water solution gradually increased (Figure 2d). When the temperature was increased from 30 °C to 50 °C at the concentration of MG in water solution as 50 mg g^−1^, the adsorption capacity of BA-CD subsequently increased a little bit. However, when the concentration of MG in water solution changed into 250 mg g^−1^, the adsorption capacity of BA-CD dramatically increased from 682.2 mg g^−1^ to 716.7 mg g^−1^ in accordance with the increase in temperature from 30 °C to 50 °C.

**Figure 7:**
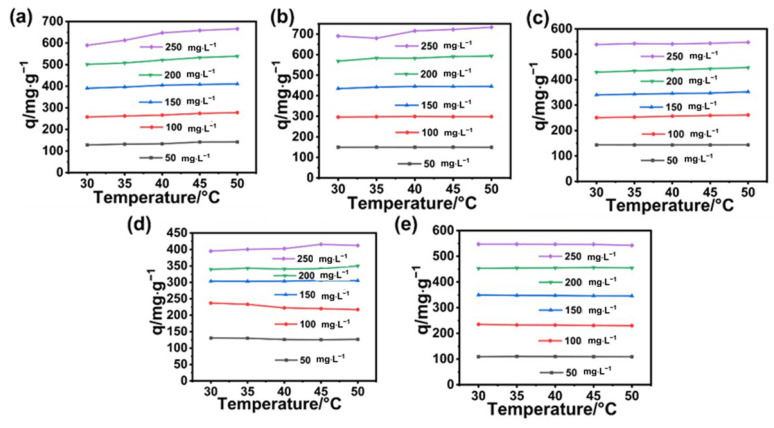
Effects of temperature on the adsorption properties of CV (**a**), RB (**b**), MB (**c**), DR (**d**), and TZ (**e**).

It was supposed that because high temperature can increase the rate of movement of dyes, the chance of dyes contacting the adsorption sites on BA-CD will also increase, leading to an increase in adsorption capacity. Interestingly, at the same temperature, the adsorption capacity of BA-CD increases as the concentration of MG increases. Furthermore, as shown in Figure 7c,e, MB and TZ showed almost no change with the variable temperatures under varying concentration. Meanwhile, the adsorption capacity of CV, RB, and DR had a slight change with the changed temperature (Figure 7a,b,d), which might be related to the characteristics of these dyes. In addition, the adsorption performance of BA-CD for anions was significantly lower than that for cations in changing both concentration and temperature.

### 3.6. Regeneration of Adsorbents

**Figure 8:**
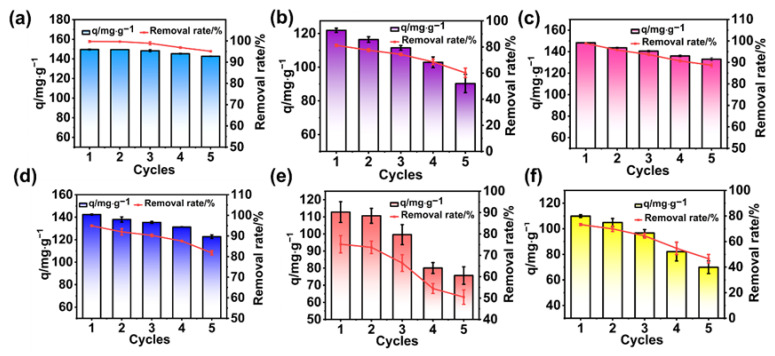
(**a**–**f**) Regeneration of MG, CV, RB, MB, DR, and TZ, respectively.

Adsorbent performance can usually be judged by reusability. We employed the previous vibration conditions and stopped the adsorption when it reached equilibrium. After filtering the adsorbent through sand filtration equipment, the adsorbent was washed with an ethanol solution in several soaks, which washed out the dyes. The adsorbent was then dried to a constant weight and the next repeated adsorption could be continuously carried out. The reusability of the adsorbent can be assessed based on the results of the repeated cycles of this experiment. From the adsorption/desorption experiments of MG dye by BA-CD (Figure 2d), it was observed that the adsorption capacity decreases with the increase in the number of cycles (Figure 8a). This might be caused by the fact that the elution method does not wash out the dye molecules completely and some of the adsorption sites are occupied, thus affecting the adsorption performance. After five cycles (pH = 7, temperature = 30 °C, conc. (MG) = 50 mg L^−1^), the removal of MG by BA-CD could reach 95%. Additionally, the removal rate of MB and RB reached more than 80%, and BA-CD also exhibited reasonable reusability for the absorbance of CV, DR, and TZ, as shown in Figure 8.

### 3.7. Comparison with Other Adsorbents

Overall, BA-CD performs very well in adsorbing diverse dyes, as discussed above; for example, the adsorption performance of BA-CD for cationic dyes was significantly higher than that for anions. Furthermore, in comparison with reported data on different adsorbents of dyes in the literature, BA-CD has some advantages in performance in the treatment of dyes (Table S1), revealing an adsorption capacity in the upper-middle range. In particular, BA-CD has the best adsorption capacity of the individual dyes such as MG, MB, RB, and CV.

### 3.8. Adsorption Kinetic Studies

**Figure 9:**
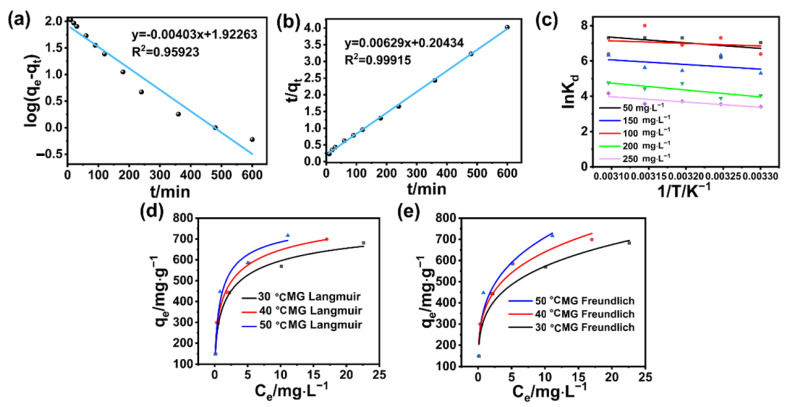
Quasi-first-order and quasi-second-order kinetic models of MG (**a**,**b**), (**c**) lnK_d_ versus 1/T curves of MG, and (**d**,**e**) Langmuir and Freundlich adsorption isotherm fitting curves of MG.

Key questions have always been raised in the study of hierarchical porous structures, such as the mechanism of recognition by diverse hierarchical pores of specific guests at the molecular level, and the relationship among these different hierarchical pores with various morphologies and characteristics [46]. With these questions in mind, the BA-CD crosslinked polymer was further employed as the hierarchical porous model with both in situ generated pores and β-CD cavity dyes to test the adsorption behavior of BA-CD by carrying out the adsorption of kinetic studies, as well as the study of adsorption isotherm (a typical example of adsorbing MG is shown in Figure 9). First, adsorption kinetics can demonstrate the adsorption process versus time. We used quasi-first-order kinetic models and quasi-second-order kinetic models to analyze the adsorption of BA-CD for various types of dyes [44,49].

(3) $$\log\left(q_{e}-q_{t}\right)=logq_{e}-\frac{K_{1}t}{2.303}$$

(4) $$\frac{t}{q_{t}}=\frac{1}{K_{2}q_{e}^{2}}+\frac{t}{q_{e}}$$

where K_1_ (min^−1^) and K_2_ (g mg^−1^ min^−1^) are the adsorption rate constants and pseudo-second-order adsorption rate constants, respectively; q_e_ (mg g^−1^) is the adsorption capacity; and q_t_ (mg g^−1^) is the adsorption capacity at a given moment.

**Figure 10:**
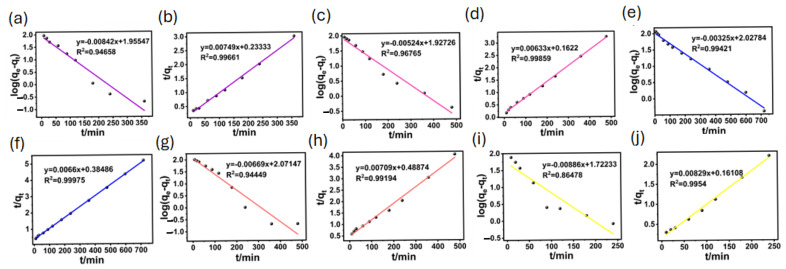
Quasi-first-order and quasi-second-order kinetic models of CV (**a**,**b**), RB (**c**,**d**), MB (**e**,**f**), DR (**g**,**h**), and TZ (**i**,**j**).

In general, from the results of the adsorption experiments, BA-CD showed the best adsorption performance for MG. MG was directly chosen as the model, and its fitted kinetic curves are shown in Figure 9a,b. The correlation parameters obtained from the fitting curves are also listed in Table 1. The adsorption data were closer to the curve of the quasi-second-order kinetic model, because its correlation coefficient was higher than that of the quasi-first-order kinetic model, leading to the obtained adsorption capacity of 158.98 mg g^−1^. This value was closer to the experimental value of 149.6 mg g^−1^. Furthermore, the adsorption process might also involve chemisorption. Interestingly, the kinetic fitting results for the other five dyes all indicated that the quasi-second-order kinetic model is more consistent with the adsorption process (Figure 10, Table S2), where the fitting results also show higher R^2^ for the quasi-second-order kinetics.

### 3.9. Study of Adsorption Isotherm

The equilibrium adsorption results were fitted using the Langmuir and Freundlich adsorption isotherm model [49] to obtain data related to the maximum adsorption capacity.

(5) $$\frac{C_{e}}{q_{e}}=\frac{C_{e}}{q_{m}}+\frac{1}{K_{L}q_{m}}$$

(6) $$\ln\left(q_{e}\right)=lnK_{F}+\frac{1}{n}ln(C_{e})$$

where C_e_ (mg L^−1^) is the equilibrium concentration, q_e_ (mg g^−1^) is the equilibrium adsorption capacity, q_m_ is the maximum adsorption capacity, K_F_ and n are constants of the Freundlich equation, and K_L_ is the constant for the Langmuir equation.

**Figure 11:**
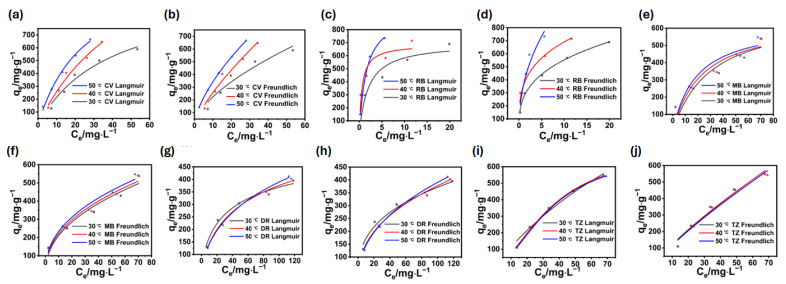
Langmuir and Freundlich adsorption isotherm fitting curves of CV (**a**,**b**), RB (**c**,**d**), MB (**e**,**f**), DR (**g**,**h**), and TZ (**i**,**j**).

The temperature change in dye adsorption by BA-CD can be described by linearity. Furthermore, the adsorption behavior was analyzed by Langmuir and Freundlich adsorption isotherm models (Figure 9d,e). Taking MG as the dye model, the correlation coefficients of the Langmuir model were higher than the parameters obtained by Freundlich. Thus, the adsorption properties of BA-CD can be further fitted by the Langmuir adsorption isotherm model, where the adsorption behavior might be monolayer adsorption. Additionally, the maximum adsorption capacities obtained at 30 °C, 40 °C, and 50 °C were 856.97 mg g^−1^, 944.099 mg g^−1^, and 821.68 mg g^−1^, respectively, indicating that the change in temperature can influence the adsorption properties. Interestingly, the correlation coefficient for Freundlich fitting is also above 0.9. The curve fitted by Freundlich can also describe the adsorption process. Thus, the 1/n value in Table 2 is less than 1, indicating that the adsorption of BA-CD is easier to carry out. Meanwhile, the K_F_ values increased gradually with the increase in temperature, indicating that this adsorption process has features of heat adsorption, in other words, elevating the temperature is favorable for such adsorption. Similarly, the simulation of adsorption isotherms of the other five dyes can also be fitted with both Langmuir and Freundlich, and the correlation coefficients’ R^2^ values are greater than 0.9 (Figure 11, Table S3).

### 3.10. Study of Adsorption Thermodynamic

Adsorption thermodynamics can determine whether the adsorption process is exothermic or absorptive, determine whether the adsorption process is spontaneous or not [49], and is important for the study of the adsorption mechanism.

(7) $$K_{d}=\frac{q_{e}}{C_{e}}$$

(8) $$lnK_{d}=\frac{∆S^{0}}{R}-\frac{∆H^{0}}{RT}$$

(9) $$∆G^{0}=∆H^{0}-T∆S^{0}$$

where K_d_ (L g^−1^) is the distribution factor, q_e_ (mg g^−1^) is the equilibrium adsorption capacity, C_e_ (mg L^−1^) is the equilibrium concentration, R (8.314 J mol^−1^ K^−1^) is the general gas constants, and T (K) is the absolute temperature.

**Figure 12:**
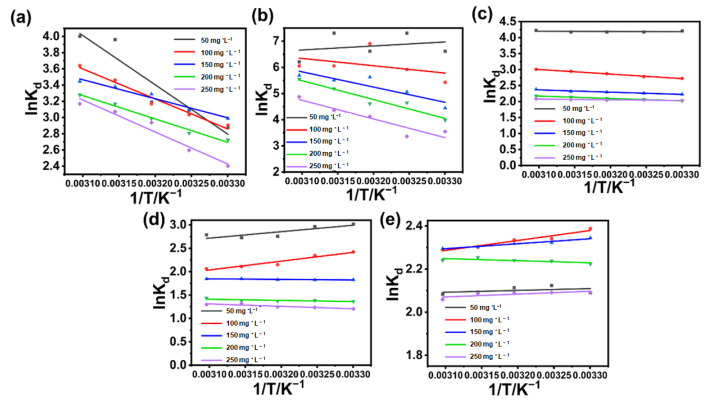
The lnK_d_ versus 1/T curves of CV (**a**), RB (**b**), MB (**c**), DR (**d**) and TZ (**e**).

The enthalpy change (ΔH), entropy change (ΔS), and Gibbs free energy (ΔG) change can be calculated based on the plotted linear regression equation graphs and the van ’t Hoff equation. All ΔG values are lower than 0, and the ΔH and ΔS values are higher than 0 (Table 3). Thus, the adsorption of the MG dye model by BA-CD at all concentrations is spontaneous, and the adsorption process is heat-absorbing, i.e., increasing the temperature promotes the adsorption process. Furthermore, the stochasticity of the solid/liquid interface during the adsorption process is also increased (Figure 9c) [47,49]. It was then found that other dyes such as CV and MB have similar fitting results to MG, with both ΔH and ΔS being greater than zero. But the left dyes including RB and TZ have the same fitting results, with ΔH and ΔS being greater than zero, indicating that elevated temperature is unfavorable for adsorption. In particular, the DR has negative values of ΔH and ΔS under specific conditions, as shown in Figure 12 and Table S4. As we discussed in our previous report [46], BA is a very small crosslinker, and after connecting two neighboring CD moieties together, these formed mono-bridged and double-bridged CD dimers can exhibit synergistic effects in capturing substrates, revealing different recognition behaviors in adsorption. But because DR has much larger molecular structures with even longer molecule length, stronger steric hindrance for BA crosslinked CDs probably weakens the synergistic effect, leading to the recorded data and possible enthalpy-driven phenomena under several conditions in Table S4.

## 4. Conclusions

Amorphous BA-CD polymers were employed in the adsorption of diverse cationic and anionic organic dyes including TZ, DR, CV, RB, MG, and MB, and they exhibited better performances in adsorption behavior in comparison with other reported macrocyclic adsorbents, as confirmed by the effects of single-factor conditions. The high adsorption capacity of BA-CD for MG increased to 716.7 mg g^−1^, with a removal rate of 95.56%. And the removal rates of the cationic and anionic dyes RB, MB, CV, DR, and TZ are 733.2 mg g^−1^, 547.2 mg g^−1^, 666.0 mg g^−1^, 412.2 mg g^−1^, and 542.4 mg g^−1^, respectively. BA-CD could undergo a continuous adsorption/desorption cycle. After five cycles, the adsorption efficiency of BA-CD on MG could reach more than 90%. In particular, we tried to answer questions raised in this study regarding adsorption mechanisms in hierarchical porous architectures, especially when fabricated by introducing macrocyclic structures such as CDs in this work. As indicated by kinetic and isothermal studies, chemical adsorption still played a significant role in the molecular recognition by BA-CD of dyes, but it was accompanied by slightly different parameters according to cases of host/guest interactions by individual β-CDs. Due to the particle sizes of the dyes and possible steric hindrance, the synergistic effect between cavities of CDs and in situ generated pores bridged by boric acid did play significant roles in the adsorption of these dyes. We believe that this study not only paves the way for adsorption of diverse cationic and anionic organic dyes, but also provides a unique strategy for fabricating β-CD-based polymeric adsorbent for the treatment of wastewater.

## Figures and Tables

**Table 1:** Relevant parameters of the two kinetic models of MG.

Kinetic Model	Parameters	Values
MG—Quasi-First-Order Kinetic Model	*K*_1_/min^−1^	0.00928
	*q_e cal_*/mg g^−1^	83.682
	R^2^	0.95923
MG—Quasi-Second-Order Kinetic Model	*K*_2_/g mg^−1^ min^−1^	0.0001936
	*q_e cal_*/mg g^−1^	158.98
	R^2^	0.99915

**Table 2:** Parameters associated with the two sorption isotherm models of MG.

Temperature/°C	Langmuir			Freundlich		
	**q_max_/mg g** **^−1^**	**K_L_/L mg** **^−1^**	**R^2^**	1/n *	**K_F_/L mg** **^−1^**	**R^2^**
30-MG	856.96948	0.69451	0.99059	0.23751	331.3781	0.97003
40-MG	944.09932	0.70368	0.98844	0.24146	367.26678	0.96459
50-MG	821.67947	0.33031	0.97055	0.26578	384.5186	0.94379

* The value of 1/n in Table 2 is related to the surface inhomogeneity of the adsorbent and could reflect the difficulty of adsorption. When 1/n is between 0 and 1, it is favorable for adsorption [49].

**Table 3:** Thermodynamically relevant parameters of MG.

Concentration of MG/mg L^−1^	Temperature/K	Δ*G*^0^/kJ mol^−1^	Δ*H*^0^/kJ mol^−1^	Δ*S*^0^/J mol^−1^ K^−1^
50-MG	303	−16.902	26.337	142.703
	313	−18.329		
	323	−19.7561		
100-MG	303	−15.7376	12.212	92.243
	313	−16.6601		
	323	−17.5825		
150-MG	303	−13.9485	21.767	117.873
	313	−15.1272		
	323	−16.306		
200-MG	303	−9.97389	32.637	140.63
	313	−11.3802		
	323	−12.7865		
250-MG	303	−8.49454	24.86	110.081
	313	−9.59535		
	323	−10.6962		

## Data Availability

The raw data supporting the conclusions of this article will be made available by the authors on request.

## Supplementary Materials

The following supporting information can be downloaded at https://www.mdpi.com/article/10.3390/polym17172357/s1, Table S1: Comparison of adsorption capacity of BA-CD with other β-CD-based materials. Table S2: Kinetic model categories and related parameters. Table S3: Parameters associated with the two sorption isotherm models. Table S4: Thermodynamically relevant parameters [50,51,52,53,54,55,56,57,58,59].
