# Supplementary material for: BA-CD Composite Polymers for Efficient Adsorption of Diverse Dyes and Its Mechanism: A Discussion-Based Thermal Dynamic and Kinetic Study
Source: Polymers (Basel). 2025 Aug 29;17(17):2357. doi: 10.3390/polym17172357 (PMC12431082; doi:10.3390/polym17172357)
Supplement: Supplementary file 1 [file polymers-17-02357-s001.zip › polymers-3807300-supplementary.pdf]

Supplementary Information

BA-CD Composite Polymers for Efficient Adsorption of Diverse Dyes and Its Mechanism Study by Thermal Dynamic and Kinetic Discussion

Zhaona Liu,[1†] Make Li[2†], Yangyang Zheng[2†], Huacheng Zhang[2*]

[1]Department of Pharmacy, Medical School, Xi'an Peihua University, Xi'an 710125, China; zhaonaliu@peihua.edu.cn

[2]School of Chemical Engineering and Technology, Xi'an Jiaotong University, Xi'an 710049, Shaanxi, P. R. China

*Corresponding author. E-mail: zhanghuacheng@xjtu.edu.cn

†Three authors contributed equally to this work.

Table S1. In comparison of adsorption capacity of BA-CD with other β-CD-based materials.

Dyes	Adsorbents	q_e (mg g^{-1})	References
MG	CD-CA/PDA	1174.67	1
MG	Fe_3O_4/β-CD/GO	990.1	2
MG	BA-CD	716.7	this work
RB	PCD-VI10	335.7	3
RB	HP-β-CD	261.78	4
RB	BA-CD	733.2	this work
MB	CD-CA/PDA	582.95	1
MB	β-Cyclodextrin–chitosan	50.12	5
MB	BA-CD	547.2	this work
CV	CD-CA/PDA	473.01	1
CV	EDTA-β-CD	104.6	6
CV	BA-CD	666.0	this work
DR	MWCNTs/Gly/β-CD	19.4	7

DR	β-CD polymer	36.2	8
DR	BA-CD	412.2	this work
TZ	Cht-PANI	397.8	9
TZ	Zn_2Al/Cl-LDH	282.48	10
TZ	BA-CD	542.4	this work

Table S2. Kinetic Model Categories and Related Parameters.

Kinetic model	Parameters	Values
CV-Quasi-First-Order Kinetic Model	K_1/min^{-1}	0.01939
	$q_{e\ cal}$/mg g^{-1}	90.24
	R^2	0.94658
CV-Quasi-Second-Order Kinetic Model	K_2/ g mg^{-1} min^{-1}	0.0002404
	$q_{e\ cal}$/mg g^{-1}	133.511
	R^2	0.99613
MB-Quasi-First-Order Kinetic Model	K_1/min^{-1}	0.00748
	$q_{e\ cal}$/mg g^{-1}	159.509
	R^2	0.99421
MB-Quasi-Second-Order Kinetic Model	K_2/ g mg^{-1} min^{-1}	0.000113
	$q_{e\ cal}$/mg g^{-1}	151.51
	R^2	0.99975
DR-Quasi-First-Order Kinetic Model	K_1/min^{-1}	0.0154
	$q_{e\ cal}$/mg g^{-1}	117.888
	R^2	0.94449
DR-Quasi-Second-Order Kinetic Model	K_2/ g mg^{-1} min^{-1}	0.0001029
	$q_{e\ cal}$/mg g^{-1}	141.0437
	R^2	0.99194
	K_1/min^{-1}	0.0204

TZ-Quasi-First-Order Kinetic Model	$q_{e\,cal}$/mg g^{-1}	52.763
	R^2	0.86478
TZ-Quasi-Second-Order Kinetic Model	K_2/ g mg^{-1} min^{-1}	0.000427
	$q_{e\,cal}$/mg g^{-1}	120.627
	R^2	0.99463
RB-Quasi-First-Order Kinetic Model	K_1/min^{-1}	0.01207
	$q_{e\,cal}$/mg g^{-1}	84.58
	R^2	0.96765
RB-Quasi-Second-Order Kinetic Model	K_2/ g mg^{-1} min^{-1}	0.000247
	$q_{e\,cal}$/mg g^{-1}	157.98
	R^2	0.99859

Table S3. Parameters associated with the two sorption isotherm models.

Isothermal Adsorption Model Categories and Related Parameters						
Temperature/℃	Langmuir			Freundlich		
	q_{max}/mg g^{-1}	K_L/L mg^{-1}	R^2	1/n	K_F/L mg^{-1}	R^2
30-CV	1050.42	0.02571	0.97157	0.61277	54. 44468	0.93723
40-CV	1644.81	0.01865	0.98171	0.75359	45.41253	0.97466
50-CV	1255.46	0.03869	0.99278	0.64683	77.15469	0.99991
30-RB	698.44601	0.50362	0.91403	0.32438	261.95962	0.99852
40-RB	687.49762	1.66463	0.93104	0.28711	356.97882	0.95351
50-RB	945.34342	0.6483	0.99412	0.44439	358.83698	0.94898
30-MB	694.53472	0.03383	0.8457	0.44091	76.10284	0.94944
40-MB	650.96635	0.04451	0.96784	0.42892	82.29029	0.96795
50-MB	636.51073	0.05371	0.9765	0.41886	88.98398	0.9766

30-DR	422.579945	0.06138	0.98701	0.33397	79.78852	0.97768
40-DR	470.95453	0.03747	0.98803	0.4005	59.70424	0.98271
50-DR	506.09479	0.03133	0.98937	0.42999	53.90678	0.98807
30-TZ	704.09238	0.00224	0.98078	0.80937	18.73306	0.97036
40-TZ	734.09237	0.00228	0.99956	0.82492	17.58287	0.97442
50-TZ	705.61366	0.00172	0.99971	0.82441	17.27832	0.96853

Table S4. Thermodynamically relevant parameters.

The Concentration of dyes/mg L^{-1}	Temperature/K	ΔG^0/kJ mol^{-1}	ΔH^0/kJ mol^{-1}	ΔS^0/J mol^{-1} K^{-1}
50-CV	303	-7.03894	50.545	190.046
	313	-8.9394		
	323	-10.8399		
100-CV	303	-7.2009	30.775	125.333
	313	-8.45423		
	323	-9.70756		
150-CV	303	-7.54732	19.533	89.374
	313	-8.44106		
	323	-9.3348		
200-CV	303	-6.7858	23.969	101.501
	313	-7.80081		
	323	-8.81582		
250-CV	303	-6.11108	32.812	128.459
	313	-7.39567		
	323	-8.68026		
50-RB	303	-17.5608	-12.688	16.082
	313	-17.7217		
	323	-17.8825		
100-RB	303	-14.5643	23.264	124.846
	313	-15.8128		
	323	-17.0613		
150-RB	303	-11.75	48.324	198.264
	313	-13.7326		
	323	-15.7153		

200-RB	303	-10.1951	59.91	231.37
	313	-12.5088		
	323	-14.8225		
250-RB	303	-8.34966	59.246	223.088
	313	-10.5805		
	323	-12.8114		
50-DR	303	-7.54127	-11.463	-12.943
	313	-7.41184		
	323	-7.28241		
100-DR	303	-6.0816	-15.667	-31.635
	313	-5.76525		
	323	-5.4489		
150-DR	303	-4.59244	0.9867	18.413
	313	-4.77657		
	323	-4.9607		
200-DR	303	-3.4273	2.107	18.265
	313	-3.60995		
	323	-3.7926		
250-DR	303	-3.03869	4.333	24.329
	313	-3.28198		
	323	-3.52527		
50-TZ	303	-5.31475	-0.704	15.217
	313	-5.46692		
	323	-5.61909		
100-TZ	303	-5.99324	-3.838	7.113
	313	-6.06437		
	323	-6.1355		
150-TZ	303	-5.89621	-1.916	13.136
	313	-6.02757		
	323	-6.15893		
200-TZ	303	-5.6156	0.808	21.2
	313	-5.8276		
	323	-6.0396		
250-TZ	303	-5.28313	-1.099	13.809
	313	-5.42122		
	323	-5.55931		
50-MB	303	-10.5506	0.4392	36.27
	313	-10.9133		

	323	-11.276		
	303	-6.84946	11.882	61.82
100-MB	313	-7.46766		
	323	-8.08586		
	303	-5.60652	5.859	37.84
150-MB	313	-5.98492		
	323	-6.36332		
	303	-5.09732	6.257	37.473
200-MB	313	-5.47205		
	323	-5.84678		
	303	-5.12323	2.010	23.542
250-MB	313	-5.35865		
	323	-5.59407		

References

1 Chen, H.; Zhou, Y.; Wang, J.; Lu, J.; Zhou, Y. Polydopamine modified cyclodextrin polymer as efficient adsorbent for removing cationic dyes and Cu^{2+}. *Journal of Hazardous Materials* **2020**, *389*, 121897.

2 Wang, D.; Liu, L.; Jiang, X.; Yu, J.; Chen, X. Adsorption and removal of malachite green from aqueous solution using magnetic β-cyclodextrin-graphene oxide nanocomposites as adsorbents. *Colloids and Surfaces A: Physicochemical and Engineering Aspects* **2015**, *466*, 166-173.

3 Qin, X.; Bai, L.; Tan, Y.; Li, L.; Song, F.; Wang, Y. β-Cyclodextrin-crosslinked polymeric adsorbent for simultaneous removal and stepwise recovery of organic dyes and heavy metal ions: Fabrication, performance and mechanisms. *Chemical Engineering Journal* **2019**, *372*, 1007-1018.

4 Maniyazagan, M.; Chakraborty, S.; Pérez-Sánchez, H.; Stalin, T. Encapsulation of triclosan within 2-hydroxypropyl–β–cyclodextrin cavity and its application in the chemisorption of rhodamine B dye. *Journal of Molecular Liquids* **2019**, *282*, 235-243.

5 Fan, L.; Luo, C.; Sun, M.; Qiu, H.; Li, X. Synthesis of magnetic β-cyclodextrin–chitosan/graphene oxide as nanoadsorbent and its application in dye adsorption and removal. *Colloids and Surfaces B: Biointerfaces* **2013**, *103*, 601-607.

6 Zhao, F.; Repo, E.; Yin, D.; Meng, Y.; Jafari, S.; Sillanpää, M. EDTA-Cross-Linked β-Cyclodextrin: An Environmentally Friendly Bifunctional Adsorbent for

Simultaneous Adsorption of Metals and Cationic Dyes. *Environmental Science & Technology* **2015**, *49* (17), 10570-10580.

7 Mohammadi, A.; Veisi, P. High adsorption performance of β-cyclodextrin-functionalized multi-walled carbon nanotubes for the removal of organic dyes from water and industrial wastewater. *Journal of Environmental Chemical Engineering* **2018**, *6* (4), 4634-4643.

8 Ozmen, E. Y.; Yilmaz, M. Use of β-cyclodextrin and starch based polymers for sorption of Congo red from aqueous solutions. *Journal of Hazardous Materials* **2007**, *148* (1-2), 303-310.

9 Sahnoun, S.; Boutahala, M. Adsorption removal of tartrazine by chitosan/polyaniline composite: Kinetics and equilibrium studies. *International Journal of Biological Macromolecules* **2018**, *114*, 1345-1353.

10 Ouassif, H.; Moujahid, E. M.; Lahkale, R.; Sadik, R.; Bouragba, F. Z.; Sabbar, E. m.; Diouri, M. Zinc-Aluminum layered double hydroxide: High efficient removal by adsorption of tartrazine dye from aqueous solution. *Surfaces and Interfaces* **2020**, *18,* 100401.
